# Comparison of Optical Coherence Tomography Angiography and Laser Speckle Flowgraphy for the Diagnosis of Normal-Tension Glaucoma

**DOI:** 10.1155/2018/1751857

**Published:** 2018-01-31

**Authors:** Asuka Takeyama, Kyoko Ishida, Ayako Anraku, Masahiro Ishida, Goji Tomita

**Affiliations:** ^1^Department of Ophthalmology, Teikyo University Hospital, Mizonokuchi, Kawasaki, Japan; ^2^Department of Ophthalmology, Toho University Ohashi Medical Center, Tokyo, Japan

## Abstract

**Purpose:**

To compare optical coherence tomography angiography (OCT-A) and laser speckle flowgraphy (LSFG) for the diagnosis of normal-tension glaucoma (NTG).

**Methods:**

Twenty-eight eyes of 28 patients with NTG and 25 eyes of 25 normal subjects matched for age, refractive errors, systemic blood pressure, and central corneal thickness were evaluated. OCT-A was used to measure whole image vessel density, inside disc vessel density, and peripapillary vessel density; using LSFG, mean blur rate (MBR) inside the whole optic nerve head (ONH) area (MBR_A_), and MBR of the vessel area (MBR_V_) and tissue area (MBR_T_) inside the ONH, were determined. Receiver operating characteristic (ROC) curves and areas under the ROC (AUROC) were used to assess the diagnostic ability of each variable.

**Results:**

The AUROC for OCT-A whole image vessel density (0.950) was significantly greater than that for OCT-A peripapillary vessel density (0.830) and for all LSFG parameters (MBR_A_ = 0.793, MBR_V_ = 0.601, and MBR_T_ = 0.61) (*P* < 0.001). The AUROC for OCT-A inside disc vessel density (0.931) was significantly greater than that for all LSFG parameters (*P* < 0.005).

**Conclusions:**

OCT-A vessel density had a higher glaucoma diagnostic ability compared to all LSFG parameters in patients with NTG.

## 1. Introduction

Knowledge about ocular blood flow is essential for understanding the pathological conditions associated with and the treatment of various ocular diseases. Blood flow in the optic nerve head (ONH) is reportedly reduced in some ocular diseases, including glaucoma [1, 2]. Besides increased intraocular pressure (IOP), a number of studies have suggested that ocular and systemic blood flow abnormalities, especially in the ONH, play an important role in the development and progression of glaucoma [3–7]. An improved understanding of ONH blood flow abnormalities would greatly assist the identification of IOP-independent factors that influence the pathophysiology of glaucoma.

Optical coherence tomography (OCT), which is able to measure retinal thickness, is commonly used in the diagnosis and management of retinal diseases and glaucoma [8–11]. In recent years, the technique of OCT angiography (OCT-A) has been developed to detect changes in OCT signals caused by flowing red blood cells in blood vessels. A newly developed OCT-A modality using split-spectrum amplitude-decorrelation angiography (SSADA) has demonstrated the ability to quantify retinal and disc blood flow rapidly and accurately [12–14]. It provides high-quality three-dimensional angiography using ultrahigh-speed OCT. Jia et al. [15] reported a reduced disc perfusion in a group of patients with early glaucoma and a link between ONH vessel density and visual field pattern standard deviation (PSD) using a custom swept-source OCT device.

Laser speckle flowgraphy (LSFG) is a noninvasive real-time method used to measure relative blood flow velocity in the ONH, retina, and choroid [16]. The main parameter of LSFG is mean blur rate (MBR), which has been reported to have high reproducibility in normal subjects as well as in glaucoma patients [17]. Previous studies have reported that MBR in the ONH tissue area was highly correlated with hydrogen gas clearance and microsphere methods in nonhuman primates [18, 19]. Moreover, MBR in normal human subjects has been found to be stable independent of age or sex [20]. Recent reports have shown that MBR is an independent factor related to retinal nerve fiber layer (RNFL) thickness and visual field indices such as mean deviation (MD) and PSD [21].

In the current study, we evaluated the relationships between vessel density obtained via OCT-A and the MBR obtained via LSFG and compared the ability of the two modalities to diagnose normal-tension glaucoma (NTG) with each other as well as with that of RNFL thickness and ganglion cell complex (GCC) thickness measurements obtained via spectral-domain- (SD-) OCT.

## 2. Materials and Methods

We retrospectively reviewed the medical records of Japanese patients with NTG who underwent LSFG and OCT-A ocular circulation measurements from November 2015 to August 2016, at the Department of Ophthalmology, Toho University Ohashi Medical Center, Tokyo, Japan. The study was approved by the Toho University Ohashi Medical Center Institutional Review Board (number H16007), and all study conduct adhered to the tenets of the Declaration of Helsinki.

Inclusion criteria were NTG defined as normal and open anterior chamber angles on slit-lamp biomicroscopy and gonioscopy, glaucomatous ONH appearance on stereoscopic evaluation with a corresponding visual field defect, intraocular pressure (IOP) ≤21 mmHg on at least three different days, best-corrected visual acuity of at least 20/25, spherical equivalent refractive errors between −6.00 and +3.00 diopters (D), and a refractive cylindrical error within 3.00 D. Subjects with any of the following were excluded from the analyses: history of intraocular surgery and intraocular eye disease other than NTG. Characteristic visual field loss was defined based on the criteria of the Anderson-Patella classification. Glaucomatous visual field loss was defined as a glaucoma hemifield test graded “outside normal limits” and a cluster of three contiguous points at the 5% level on a pattern deviation plot, using the threshold test strategy with the Humphrey Field Analyzer (Humphrey-Zeiss Systems, Dublin, CA, USA) 30-2 Swedish Interactive Threshold Algorithm standard automated perimetry. Patients with mean deviation (MD) value of visual field global indices < −15 dB were excluded from the study.

The inclusion criteria for normal subjects were a normal-appearing ONH, intact neuroretinal rim and RNFL, normal standard automated perimetry (defined as a glaucoma hemifield test within normal limits and a PSD within 95% confidence interval limits), and IOP of ≤21 mmHg. Patients with diabetic retinopathy and other diseases that may cause visual field loss or optic disc abnormalities, an unreliable visual field test (>15% false positives or false negatives or >20% fixation losses), history of intraocular surgery or patients for which there was an inability to clinically view or photograph the optic discs due to media opacity or poorly dilating pupils were excluded.

Both groups were matched for age, spherical equivalent refractive errors, systemic blood pressure, and central corneal thickness (CCT).

### 2.1. Clinical Examinations

For all subjects, age, gender, best-corrected visual acuity on a logarithmic scale, slit-lamp biomicroscopy, IOP, CCT, and stereoscopic fundus examination results were recorded. Noncycloplegic refraction was measured with an auto ref-keratometer (ARK-530A; Nidek, Aichi, Japan). The refraction data were converted to the spherical equivalent, which was calculated using the spherical diopter (D) plus one half of the cylindrical dioptric power.

IOP was measured using a Goldmann applanation tonometer on the same day the LSFG and OCT-A were measured. Systolic blood pressure (SBP) and diastolic blood pressure (DBP) were measured before performing LSFG measurements. Mean blood pressure (MBP) and mean ocular perfusion pressure (MOPP) were calculated.

In all subjects, OCT-A and LSFG were performed simultaneously on the same day, and visual field was measured within 6 months.

### 2.2. SD-OCT Measurements

Mean RNFL and GCC thicknesses were measured using the RTVue-XR Avanti system (Optovue Inc., Fremont, CA, USA). This system uses a scanning laser diode that emits a scan beam of 840 nm ± 10 nm and provides images of ocular microstructures. The ONH scanning protocol was used for measuring the RNFL thickness. The total time for each single scan acquisition was 0.55 second. Using dedicated software, RNFL thickness was automatically measured at a diameter of 3.45 mm around the center of the optic disc. A total of 775 A-scans were obtained at this circumference. The RNFL thickness parameter was designed to evaluate the mean thickness of a 360-degree area. In this study, the GCC scanning protocol was used to determine GCC thickness, which was measured from the inner limiting membrane to the outer retinal pigment epithelium. The GCC protocol consisted of 15 vertical line scans covering a 7 × 7 mm square region. To achieve the best coverage possible within the temporal region, the GCC protocol scan was centered at 1 mm temporal to the fovea center. During the total scanning period, the GCC protocol captured 15,000 data points within 0.6 second. Only good-quality images with a signal strength index of 40 or more and without segmentation failure or artifacts were included.

### 2.3. OCT-A Measurements

OCT was performed with the RTVue-XR Avanti with AngioVue software (Optovue Inc., version 2015.100.0.35). After pupil dilation via the administration of 0.4% tropicamide to the eye over a 20-minute period, a well-trained operator obtained good-quality OCT images. Images were excluded if the signal strength indicator was <40 owing to media opacity, patient positioning, or excessive eye movement. The instrument used for OCT-A images was based on the AngioVue Imaging System for obtaining amplitude decorrelation angiography images. The instrument has an A-scan rate of 70,000 per second using a light source centered on 840 nm and a bandwidth of 50 nm. Each OCT-A volume contains 304 × 304 A-scans with two consecutive B-scans captured at each fixed position before proceeding to the next sampling location. SSADA was employed to improve the signal-to-noise ratio by splitting the spectrum to generate multiple repeat OCT frames from the two original repeat OCT frames [13].

The software analyzes the amplitude of variation of the OCT signal over time for every location acquired and calculates decorrelation. Static tissue yields low decorrelation values as the signal varies, because moving red blood cells cross the OCT beam and cause the signal amplitude to vary rapidly over time. A threshold decorrelation value is therefore used to discriminate blood flow from static tissue. The angiography information displayed was the average of the decorrelation values when viewed perpendicularly through the thickness evaluated. This maps all the detected vessels in the acquired volume.

The Angio Disc protocol uses a flow density analysis tool for the optic disc and peripapillary retina. The inside of the circle is elliptical to fit the ONH margin that appears in the OCT enface image. Whole enface image vessel density was measured in the entire 4.5 × 4.5 mm image, and peripapillary vessel density was calculated in the region defined as a 750 *μ*m wide elliptical annulus extending from the optic disc boundary, using the dedicated software provided by Optovue (AngioAnalytics™). Segmentation algorithms used in the surface mode included signals from the inner limiting membrane to the nerve fiber layer. Vessel density was defined as the percentage area occupied by the vessel, as measured using the intensity-based thresholding feature of the software, which adopted the same method of calculation as previously reported [13].

### 2.4. LSFG Measurements

The principles of LSFG have been described in detail previously [16]. Briefly, the instrument consists of a fundus camera equipped with a diode laser (wavelength 830 nm) and an ordinary charge-coupled device camera (resolution 750 × 360 pixels). In the current study, ocular circulation was evaluated with LSFG software (LSFG-NAVI, version 3.1.39.2, Softcare Ltd., Fukuoka, Japan), and MBR was used as a relative measure of blood flow. All examinations were performed by a single experienced operator. Optic disc edge detection in MBR images was manually marked on the image, and the disc edge location was saved by the software. Vessels were then automatically segmented by the system's software (LSFG Analyzer, version 3.0.47.0) using an automated defining threshold. Mean MBR (MBR_A_), MBR in the vessel area (MBR_V_), and MBR in the tissue area (MBR_T_) values were generated. Subjects' pupils were dilated using 0.4% tropicamide (Mydrin-M; Santen Pharmaceutical Co. Ltd., Osaka, Japan) before LSFG examination, and three consecutive measurements were taken for each subject. The average of the three measurements was used in the analyses.

### 2.5. Statistical Analysis

The Mann–Whitney *U* test was used to compare pairs of independent groups, and the chi-square test was used to analyze categorical data. Spearman's rank correlation coefficients were calculated to evaluate the relationships between the OCT-A and LSFG measurements. Receiver operating characteristic (ROC) curves were used to assess the ability of each of the variables to differentiate glaucomatous eyes from normal eyes. ROC curves show the trade-off between sensitivity and specificity. An area under the ROC curve (AUROC) of 1.0 represents perfect discrimination, whereas an AUROC of 0.5 represents chance discrimination. MedCalc version 16.8 (MedCalc Software, Mariakerke, Belgium) was used to generate and compare ROC curves. The other statistical analyses were performed using IBM SPSS Statistics for Windows (version 19.0; SPSS, Chicago, IL, USA). Probability values less than 0.05 were considered statistically significant.

## 3. Results

Initially, 32 NTG patients and 27 normal participants were enrolled in the study. However, 4 NTG patients and 2 normal participants were subsequently excluded due to poor-quality OCT-A images and signal strengths. Thus, the final analysis included 28 eyes of 28 NTG patients (13 men, 15 women) and 25 eyes of 25 normal participants (14 men, 11 women). The characteristics of the two groups are presented in Table 1. There were no significant differences in age, sex, refraction, CCT, systemic variables (SBP, DBP, and MBP), or ocular perfusion pressure between the normal participants and glaucoma patients (*P* values ranged from 0.052 to 0.843). IOP was significantly lower in NTG patients than in normal participants (*P* = 0.016). Compared to the normal controls, the NTG eyes demonstrated visual field loss with lower MD values and higher PSD values (*P* < 0.001).

A summary of OCT-A results for vessel density, LSFG results for MBR, and SD-OCT results for GCC thickness and RNFL thickness is presented in Table 2. All vessel density, GCC, and RNFL measurements were significantly lower in the NTG group than in the normal group. There was a significant decrease in MBR_A_ in the LSFG measurements in the NTG group compared to the normal group (*P* < 0.001), but no significant decreases were observed in MBR_V_ (*P* = 0.209) or MBR_T_ (*P* = 0.212).

Table 3 shows correlations between OCT-A, LSFG, and SD-OCT parameters in the NTG patients. OCT-A whole image vessel density was significantly correlated with LSFG MBR_V_ (*r* = 0.388, *P* = 0.041) and MBR_T_ (*r* = 0.495, *P* = 0.007). OCT-A whole image vessel density was also correlated with structural variables, that is, GCC thickness (*r* = 0.710, *P* < 0.001) and RNFL thickness (*r* = 0.725, *P* < 0.001). OCT-A peripapillary vessel density was significantly correlated with GCC thickness (*r* = 0.640, *P* < 0.001) and RNFL thickness (*r* = 0.758, *P* < 0.001). OCT-A inside disc vessel density was not significantly correlated with any LSFG or SD-OCT parameters.

**Figure 1:**
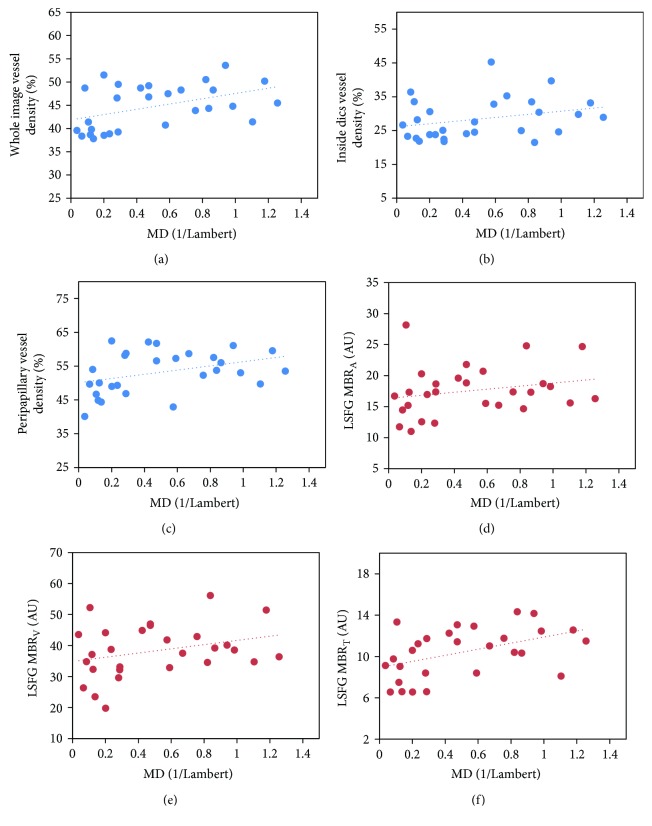
Scatter plots of MD by OCT-A whole image vessel density (a), inside disc vessel density (b), peripapillary vessel density (c), LSFG MBR_A_ (d), LSFG MBR_V_ (e), and LSFG MBR_T_ (f). MD = mean deviation; OCT-A = optical coherence tomography angiography; LSFG = laser speckle flowgraphy; MBR_A_ = mean blur rate inside the whole optic nerve head area; MBR_V_ = mean blur rate of the vessel area inside the optic nerve head area; MBR_T_ = mean blur rate of the tissue area inside the optic nerve head.

Table 4 shows the correlations between the MD value of the visual field and each of the parameters in NTG patients. OCT-A whole image vessel density (*r* = 0.468) and peripapillary vessel density (*r* = 0.403) were significantly correlated with MD in NTG patients (Figure 1). Of the LSFG parameters, only MBR_T_ (*r* = 0.460) was significantly correlated with MD. Neither age nor refraction was correlated with MD.

**Figure 2:**
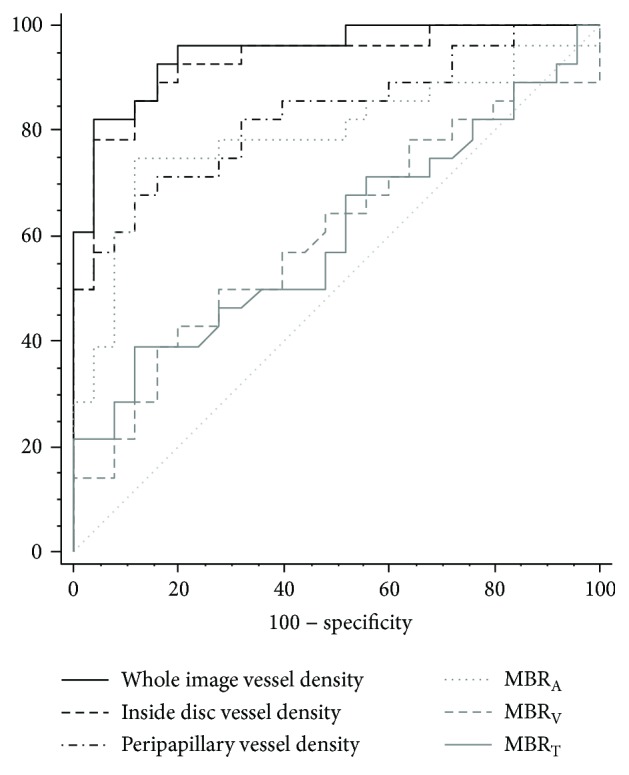
Receiver operating characteristic curves for OCT-A whole image vessel density, inside disc vessel density, peripapillary vessel density, and LSFG MBR_A_, MBR_T_, and MBR_V_. OCT-A = optical coherence tomography angiography; LSFG = laser speckle flowgraphy; MBR_A_ = mean blur rate inside the whole optic nerve head area; MBR_T_ = mean blur rate of the tissue area inside the optic nerve head; MBR_V_ = mean blur rate of the vessel area inside the optic nerve head area.

Table 5 summarizes the AUROCs for OCT-A, LSFG, and SD-OCT parameters. The AUROC for GCC thickness was the largest (0.970), followed by that for OCT-A whole vessel density (0.950) and then OCT-A inside disc vessel density (0.931). The AUROC for OCT-A whole image vessel density was significantly greater than that for OCT-A peripapillary vessel density and all the LSFG parameters (*P* < 0.001). OCT-A inside disc vessel density had a significantly greater AUROC than all the LSFG parameters (*P* < 0.005). While the AUROC for OCT-A peripapillary vessel density was significantly greater than that for LSFG MBR_V_ and MBR_T_ (*P* < 0.005), it was significantly lower than that for SD-OCT GCC (*P* < 0.005). Of the parameters with a specificity of >90%, the one with the highest sensitivity was GCC thickness, followed by OCT-A whole image vessel density. The ROC curves for the six parameters are shown in Figure 2.

## 4. Discussion

The current study compared the diagnostic ability of OCT-A with that of LSFG in patients with NTG. OCT-A whole image vessel density had higher diagnostic ability for NTG than that of all the LSFG parameters investigated, and it was equivalent to the diagnostic ability of GCC thickness.

Since OCT-A has been introduced into the clinic, a number of reports on glaucoma diagnosis via OCT-A have been published [14, 15, 22–32]. Wang et al. [23] found that the power of disc flow index and vessel density for differentiating normal eyes from eyes with open-angle glaucoma was 0.82 and 0.80, respectively, in terms of AUROC. Liu et al. [27] reported the diagnostic ability of peripapillary vessel density in 12 glaucomatous eyes and 12 normal eyes. The AUC (0.94), sensitivity (83.3%), and specificity (91.7%) of average peripapillary vessel density were comparable to those of the average RNFL thickness (0.97, 91.7%, and 91.7%, resp.). Although the diagnostic ability of peripapillary vessel density reported in their study was higher than that in our study, this may be due to the different sample sizes. Yarmohammadi et al. [24] compared mean whole image vessel density, average peripapillary vessel density, and RNFL thickness for the ability to diagnose glaucoma. In pairwise comparisons, the AUROC for whole image vessel density was significantly greater than that for peripapillary vessel density (0.94 versus 0.83). The AUROC for peripapillary vessel density measurements was smaller than that for mean RNFL thickness (0.92); however, that difference was not statistically significant. In the current study, the diagnostic abilities of average vessel density and RNFL thickness were similar to those reported by Yarmohammadi et al. [24], although the difference between the diagnostic abilities of average vessel density and average RNFL thickness was statistically significant. In addition, in the current study, whole image vessel density exhibited better diagnostic accuracy than peripapillary vessel density (0.95 versus 0.83). The reason why whole image vessel density exhibited better diagnostic ability than peripapillary vessel density is not clear. However, a larger measurement area may be advantageous for detecting changes in the radial peripapillary capillary. Similar to our results, Rao et al. [30] found that the AUCs of several OCT parameters (peripapillary RNFL thickness, 0.95; GCC thickness, 0.93) in 113 eyes with POAG and 78 normal eyes were significantly better than those of the corresponding vessel densities (whole enface vessel density, 0.93; inside disc vessel density, 0.77; and peripapillary vessel density, 0.85).

On the other hand, regarding LSFG, Shiga et al. [21] showed that the LSFG MBR_T_ of the ONH was significantly correlated with RNFL thickness, MD, and PSD of the visual field indices. Their findings supported previous reports that reduced MBR in the ONH was significantly associated with structural and visual field damage in glaucomatous eyes [1, 2, 33]. In the current study, LSFG MBR_T_ was also significantly correlated with RNFL thickness, MD, and PSD. Reported AUCs for LSFG measurements reportedly range from 0.80 to 0.89 [33–35]. Aizawa et al. [33] reported that the diagnostic ability of MBR (AUC = 0.86) and RNFL thickness (AUC = 0.91) was statistically similar (*P* = 0.25). However, in the current study, the diagnostic ability of mean MBR in terms of MBR_A_ (AUC = 0.79) was significantly lower than that of RNFL thickness (AUC = 0.91, *P* < 0.05). The reason for this discrepancy may lie in the fact that the former study included patients with relatively advanced stage glaucoma, while in the current study, the included patients showed a relatively early stage of NTG (MD [mean ± standard deviation] = −4.6 ± 4.2 [dB] and 0.5 ± 0.4 [1/Lambert]). According to our results, LSFG may be more useful for pulse waveform analysis than for NTG diagnosis [34, 36–38].

The present study had several limitations. First, its relatively small sample size and the inclusion of the participants of a single ethnicity might have influenced the results. Larger, prospective studies are needed in future to confirm the findings of the current study. Second, a potential bias might have been introduced in the current study by the various topical antiglaucoma treatments used by the patients. Approximately 70% of the NTG patients were treated and received, on average, one to three active agents (Table 1). There are no reports to date on the effects of topical antiglaucoma medications on vessel density. Thus, it is necessary to validate our results in a group of NTG patients not undergoing glaucoma treatments. Another possible limitation of the current study was that in the NTG participants, we did not consider histories of systemic hypertension or systemic antihypertensive medication use, which might have influenced blood flow measurements, although both OCTA and LSFG were performed under the same conditions.

In conclusion, we found that OCT-A whole image vessel density had a higher ability to diagnose NTG than any of the LSFG parameters investigated, and its diagnostic ability was equivalent to that of GCC thickness.

## Figures and Tables

**Table 1:** Demographics and ocular characteristics of normal subjects and normal-tension glaucoma patients.

	Normal (*N* = 25)	Normal-tension glaucoma (*N* = 28)	*P* value
Demographic characteristics			
Gender (male/female)	14/11	13/15	0.586^∗^
Laterality (right/left)	19/6	15/13	0.151^∗^
Age (years)	49.3 ± 10.0	53.7 ± 12.2	0.130^∗∗^
Clinical characteristics			
SBP (mmHg)	119.2 ± 12.1	117.4 ± 13.9	0.762^∗∗^
DBP (mmHg)	77.1 ± 9.1	72.8 ± 11.6	0.151^∗∗^
MBP (mmHg)	91.1 ± 9.4	87.6 ± 11.9	0.318^∗∗^
MOPP (mmHg)	46.2 ± 5.7	45.3 ± 7.2	0.838^∗∗^
Heart rate (beats/min)	73.3 ± 9.8	72.8 ± 11.4	0.843^∗∗^
Hypertension medications	0 (0%)	5 (17.9%)	<0.001^∗∗^
Topical glaucoma medications	0 (0%)	20 (71.4%)	<0.001^∗∗^
Prostaglandin analogues	—	18 (64.3%)	
Beta-adrenoceptor antagonists	—	3 (10.7%)	
Carbonic anhydrase inhibitors	—	2 (7.1%)	
Aloha-1-antagonists	—	1 (3.6%)	
Ocular characteristics			
Spherical equivalent (D)	−1.5 ± 1.9	−2.7 ± 2.0	0.052^∗∗^
IOP (mmHg)	14.6 ± 2.0	13.1 ± 2.7	0.016^∗∗^
CCT (*μ*m)	536.0 ± 24.8	525.3 ± 36.2	0.277^∗∗^
MD (dB)	−0.3 ± 1.5	−4.6 ± 4.2	<0.001^∗∗^
MD (1/Lambert)	1.0 ± 0.3	0.5 ± 0.4	<0.001^∗∗^
PSD (dB)	1.6 ± 0.5	7.6 ± 4.6	<0.001^∗∗^
PSD (1/Lambert)	1.5 ± 0.2	11.0 ± 15.7	<0.001^∗∗^

The data are given as mean ± SD. ^∗^The difference between groups was analyzed using *χ*^2^ test. ^∗∗^The difference between groups was determined using analysis of variance with the Mann–Whitney test. SBP: systolic blood pressure; DBP: diastolic blood pressure; MBP: mean blood pressure; MOPP: mean ocular perfusion pressure; IOP: intraocular pressure; CCT: central cornea thickness; MD: mean deviation; PSD: pattern standard deviation.

**Table 2:** Result of diagnostic testing.

	Normal (*N* = 25)	Normal-tension glaucoma (*N* = 28)	*P* value
OCT-A whole image vessel density (%)	52.89 ± 2.25	44.73 ± 4.80	<0.001^∗^
OCT-A inside disc vessel density (%)	43.08 ± 7.16	28.43 ± 6.00	<0.001^∗^
OCT-A peripapillary vessel density (%)	60.02 ± 2.90	53.23 ± 6.26	<0.001^∗^
LSFG MBR_A_ (AU)	21.01 ± 2.82	17.58 ± 3.99	<0.001^∗^
LSFG MBR_V_ (AU)	40.67 ± 5.44	38.29 ± 8.44	0.209
LSFG MBR_T_ (AU)	11.35 ± 1.62	10.42 ± 2.40	0.212
GCC thickness(*μ*m)	96.26 ± 5.56	78.12 ± 8.29	<0.001^∗^
RNFL thickness (*μ*m)	93.92 ± 7.75	77.64 ± 10.81	<0.001^∗^

Differences between groups were tested with the Mann–Whitney *U* test. The asterisk indicates statistically significance (*P* < 0.05). OCT-A: optical coherence tomography angiography; LSFG: laser speckle flowgraphy; MBR: mean blur rate; MBR_A_: mean MBR in all areas; MBR_V_: mean MBR in the vessel area; MBR_T_: mean MBR in the tissue area; AU: arbitrary units; GCC: ganglion cell complex; RNFL: retinal nerve fiber layer.

**Table 3:** Correlations between optical coherence tomography angiography parameter, laser speckle flowgraphy parameters, and spectral-domain optical coherence tomography parameters in normal tension-glaucoma patients.

	OCT-A inside disc vessel density		OCT-A peripapillary vessel density		LSFG MBR_A_		LSFG MBR_V_		LSFG MBR_T_		GCC thickness		RNFL thickness	
	*r*	*P*	*r*	*P*	*r*	*P*	*r*	*P*	*r*	*P*	*r*	*P*	*r*	*P*
OCT-A whole image vessel density	0.333	0.083	0.904	<0.001^∗^	0.374	0.050	0.388	0.041^∗^	0.495	0.007^∗^	0.710	<0.001^∗^	0.725	<0.001^∗^
OCT-A inside disc vessel density			0.109	0.581	−0.001	0.994	0.093	0.638	0.186	0.342	0.083	0.675	−0.022	0.911
OCT-A peripapillary vessel density					0.271	0.163	0.265	0.172	0.354	0.065	0.640	<0.001^∗^	0.758	<0.001^∗^
LSFG MBR_A_							0.831	<0.001^∗^	0.823	<0.001^∗^	0.531	0.004^∗^	0.573	0.001^∗^
LSFG MBR_V_									0.805	<0.001^∗^	0.517	0.005^∗^	0.535	0.003^∗^
LSFG MBR_T_											0.591	0.001^∗^	0.593	0.001^∗^
GCC thickness													0.797	<0.001^∗^

*r*: Spearman's rank coefficient of correlation. ^∗^*P* < 0.05. OCT-A: optical coherence tomography angiography; LSFG: laser speckle flowgraphy; MBR: mean blur rate; MBR_A_: mean MBR in all areas; MBR_V_: mean MBR in the vessel area; MBR_T_: mean MBR in the tissue area; GCC: ganglion cell complex; RNFL: retinal nerve fiber layer.

**Table 4:** Correlation between the visual field mean deviations and the diagnostic parameters.

	*r*	*P*
OCT-A whole image vessel density	0.468	0.012^∗^
OCT-A inside disc vessel density	0.259	0.184
OCT-A peripapillary vessel density	0.403	0.034^∗^
LSFG MBR_A_	0.295	0.127
LSFG MBR_V_	0.244	0.210
LSFG MBR_T_	0.460	0.014^∗^
GCC thickness	0.403	0.033^∗^
RNFL thickness	0.353	0.065

*r*: Spearman's rank coefficient of correlation. ^∗^*P* < 0.05. OCT-A: optical coherence tomography angiography; LSFG: laser speckle flowgraphy; MBR: mean blur rate; MBR_A_: mean MBR in all areas; MBR_V_: mean MBR in the vessel area; MBR_T_: mean MBR in the tissue area; GCC: ganglion cell complex; RNFL: retinal nerve fiber layer

**Table 5:** Comparison of parameters using the area under receiver operating characteristics curve and sensitivities at fixed specificities.

	Normal versus normal-tension glaucoma	
	AUROC (SE)	Sn/Sp (Sp > 90%)
OCT-A whole image vessel density	0.950 (0.027)	82.14/92.00
OCT-A inside disc vessel density	0.931 (0.034)	78.57/92.00
OCT-A peripapillary vessel density	0.830 (0.056)^∗^	60.71/92.00
LSFG MBR_A_	0.793 (0.065)^∗†^	60.71/92.00
LSFG MBR_V_	0.601 (0.079)^∗†‡^	21.43/92.00
LSFG MBR_T_	0.601 (0.079)^∗†‡^	28.57/92.00
GCC thickness	0.970 (0.021)^‡^	92.86/92.00
RNFL thickness	0.906 (0.039)	75.00/96.00

^∗^ *P* < 0.01 for comparison of OCT-A whole image vessel density. ^†^*P* < 0.05 for comparison of OCT-A inside disc vessel density. ^‡^*P* < 0.05 for comparison of OCT-A peripapillary vessel density. AUROC: area under receiver operating characteristic curve; SE: standard error; OCT-A: optical coherence tomography angiography; LSFG: laser speckle flowgraphy; MBR: mean blur rate; MBR_A_: mean MBR in all areas; MBR_V_: mean MBR in the vessel area; MBR_T_: mean MBR in the tissue area; GCC: ganglion cell complex; RNFL: retinal nerve fiber layer; Sn: sensitivity; Sp: specificities.

## References

[1] Chiba N., Omodaka K., Yokoyama Y. (2011). Association between optic nerve blood flow and objective examinations in glaucoma patients with generalized enlargement disc type. *Clinical Ophthalmology*.

[2] Yokoyama Y., Aizawa N., Chiba N. (2011). Significant correlations between optic nerve head microcirculation and visual field defects and nerve fiber layer loss in glaucoma patients with myopic glaucomatous disk. *Clinical Ophthalmology*.

[3] Leske M. C., Connell A. M., SY W., Hyman L. G., Schachat A. P. (1995). Risk factors for open-angle glaucoma. The Barbados eye study. *Archives of Ophthalmology*.

[4] Flammer J., Orgul S. (1998). Optic nerve blood-flow abnormalities in glaucoma. *Progress in Retinal and Eye Research*.

[5] Bonomi L., Marchini G., Marraffa M., Bernardi P., Morbio R., Varotto A. (2000). Vascular risk factors for primary open angle glaucoma: the Egna-Neumarkt Study. *Ophthalmology*.

[6] Mitchell P., Lee A. J., Rochtchina E., Wang J. J. (2004). Open-angle glaucoma and systemic hypertension: the blue mountains eye study. *Journal of Glaucoma*.

[7] Caprioli J., Coleman A. L., Blood Flow in Glaucoma Discussion (2010). Blood pressure, perfusion pressure, and glaucoma. *American Journal of Ophthalmology*.

[8] Puliafito C. A., Hee M. R., Lin C. P. (1995). Imaging of macular diseases with optical coherence tomography. *Ophthalmology*.

[9] Hee M. R., Puliafito C. A., Wong C. (1995). Optical coherence tomography of macular holes. *Ophthalmology*.

[10] Schuman J. S., Hee M. R., Arya A. V. (1995). Optical coherence tomography: a new tool for glaucoma diagnosis. *Current Opinion in Ophthalmology*.

[11] Schuman J. S., Hee M. R., Puliafito C. A. (1995). Quantification of nerve fiber layer thickness in normal and glaucomatous eyes using optical coherence tomography. *Archives of Ophthalmology*.

[12] Jia Y., Morrison J. C., Tokayer J. (2012). Quantitative OCT angiography of optic nerve head blood flow. *Biomedical Optics Express*.

[13] Jia Y., Tan O., Tokayer J. (2012). Split-spectrum amplitude-decorrelation angiography with optical coherence tomography. *Optics Express*.

[14] Wang X., Jia Y., Spain R. (2014). Optical coherence tomography angiography of optic nerve head and parafovea in multiple sclerosis. *The British Journal of Ophthalmology*.

[15] Jia Y., Wei E., Wang X. (2014). Optical coherence tomography angiography of optic disc perfusion in glaucoma. *Ophthalmology*.

[16] Sugiyama T., Araie M., Riva C. E., Schmetterer L., Orgul S. (2010). Use of laser speckle flowgraphy in ocular blood flow research. *Acta Ophthalmologica*.

[17] Aizawa N., Yokoyama Y., Chiba N. (2011). Reproducibility of retinal circulation measurements obtained using laser speckle flowgraphy-NAVI in patients with glaucoma. *Clinical Ophthalmology*.

[18] Takahashi H., Sugiyama T., Tokushige H. (2013). Comparison of CCD-equipped laser speckle flowgraphy with hydrogen gas clearance method in the measurement of optic nerve head microcirculation in rabbits. *Experimental Eye Research*.

[19] Wang L., Cull G. A., Piper C., Burgoyne C. F., Fortune B. (2012). Anterior and posterior optic nerve head blood flow in nonhuman primate experimental glaucoma model measured by laser speckle imaging technique and microsphere method. *Investigative Ophthalmology & Visual Science*.

[20] Aizawa N., Kunikata H., Nitta F. (2016). Age- and sex-dependency of laser speckle flowgraphy measurements of optic nerve vessel microcirculation. *PLoS One*.

[21] Shiga Y., Kunikata H., Aizawa N. (2016). Optic nerve head blood flow, as measured by laser speckle flowgraphy, is significantly reduced in preperimetric glaucoma. *Current Eye Research*.

[22] Lee E. J., Lee K. M., Lee S. H., Kim T. W. (2016). OCT angiography of the peripapillary retina in primary open-angle glaucoma. *Investigative Ophthalmology & Visual Science*.

[23] Wang X., Jiang C., Ko T. (2015). Correlation between optic disc perfusion and glaucomatous severity in patients with open-angle glaucoma: an optical coherence tomography angiography study. *Graefe's Archive for Clinical and Experimental Ophthalmology*.

[24] Yarmohammadi A., Zangwill L. M., Diniz-Filho A. (2016). Optical coherence tomography angiography vessel density in healthy, glaucoma suspect, and glaucoma eyes. *Investigative Ophthalmology & Visual Science*.

[25] Yarmohammadi A., Zangwill L. M., Diniz-Filho A. (2016). Relationship between optical coherence tomography angiography vessel density and severity of visual field loss in glaucoma. *Ophthalmology*.

[26] Leveque P. M., Zeboulon P., Brasnu E., Baudouin C., Labbé A. (2016). Optic disc vascularization in glaucoma: value of spectral-domain optical coherence tomography angiography. *Journal of Ophthalmology*.

[27] Liu L., Jia Y., Takusagawa H. L. (2015). Optical coherence tomography angiography of the peripapillary retina in glaucoma. *JAMA Ophthalmology*.

[28] Rao H. L., Kadambi S. V., Weinreb R. N. (2016). Diagnostic ability of peripapillary vessel density measurements of optical coherence tomography angiography in primary open-angle and angle-closure glaucoma. *The British Journal of Ophthalmology*.

[29] Rao H. L., Pradhan Z. S., Weinreb R. N. (2016). Regional comparisons of optical coherence tomography angiography vessel density in primary open-angle glaucoma. *American Journal of Ophthalmology*.

[30] Rao H. L., Pradhan Z. S., Weinreb R. N. (2017). A comparison of the diagnostic ability of vessel density and structural measurements of optical coherence tomography in primary open angle glaucoma. *PLoS One*.

[31] Akagi T., Iida Y., Nakanishi H. (2016). Microvascular density in glaucomatous eyes with hemifield visual field defects: an optical coherence tomography angiography study. *American Journal of Ophthalmology*.

[32] Chihara E., Dimitrova G., Amano H., Chihara T. (2017). Discriminatory power of superficial vessel density and prelaminar vascular flow index in eyes with glaucoma and ocular hypertension and normal eyes. *Investigative Ophthalmology & Visual Science*.

[33] Aizawa N., Kunikata H., Shiga Y., Yokoyama Y., Omodaka K., Nakazawa T. (2014). Correlation between structure/function and optic disc microcirculation in myopic glaucoma, measured with laser speckle flowgraphy. *BMC Ophthalmology*.

[34] Shiga Y., Omodaka K., Kunikata H. (2013). Waveform analysis of ocular blood flow and the early detection of normal tension glaucoma. *Investigative Ophthalmology & Visual Science*.

[35] Maekawa S., Shiga Y., Kawasaki R., Nakazawa T. (2014). Usefulness of novel laser speckle flowgraphy-derived variables of the large vessel area in the optic nerve head in normal tension glaucoma. *Clinical & Experimental Ophthalmology*.

[36] Shiba T., Takahashi M., Hori Y., Maeno T. (2012). Pulse-wave analysis of optic nerve head circulation is significantly correlated with brachial–ankle pulse-wave velocity, carotid intima–media thickness, and age. *Graefe's Archive for Clinical and Experimental Ophthalmology*.

[37] Shiba T., Takahashi M., Maeno T. (2014). Pulse-wave analysis of optic nerve head circulation is significantly correlated with kidney function in patients with and without chronic kidney disease. *Journal of Ophthalmology*.

[38] Shiba T., Takahashi M., Hashimoto R., Matsumoto T., Hori Y. (2016). Pulse waveform analysis in the optic nerve head circulation reflects systemic vascular resistance obtained via a Swan–Ganz catheter. *Graefe's Archive for Clinical and Experimental Ophthalmology*.

